# Whispers in the Blood: Leveraging MicroRNAs for Unveiling Autologous Blood Doping in Athletes

**DOI:** 10.3390/ijms25010249

**Published:** 2023-12-23

**Authors:** Mehdi Hassanpour, Amankeldi A. Salybekov

**Affiliations:** Qazaq Institute of Innovative Medicine, Regenerative Medicine Division, Cell and Gene Therapy Department, Astana 020000, Kazakhstan

**Keywords:** autologous blood doping, biomarker detection, extracellular vesicles, microRNAs

## Abstract

The prevalence of autologous blood transfusions (ABTs) presents a formidable challenge in maintaining fair competition in sports, as it significantly enhances hemoglobin mass and oxygen capacity. In recognizing ABT as a prohibited form of doping, the World Anti-Doping Agency (WADA) mandates stringent detection methodologies. While current methods effectively identify homologous erythrocyte transfusions, a critical gap persists in detecting autologous transfusions. The gold standard practice of longitudinally monitoring hematological markers exhibits promise but is encumbered by limitations. Despite its potential, instances of blood doping often go undetected due to the absence of definitive verification processes. Moreover, some cases remain unpenalized due to conservative athlete-sanctioning approaches. This gap underscores the imperative need for a more reliable and comprehensive detection method capable of unequivocally differentiating autologous transfusions, addressing the challenges faced in accurately identifying such prohibited practices. The development of an advanced detection methodology is crucial to uphold the integrity of anti-doping measures, effectively identifying and penalizing instances of autologous blood transfusion. This, in turn, safeguards the fairness and equality essential to competitive sports. Our review tackles this critical gap by harnessing the potential of microRNAs in ABT doping detection. We aim to summarize alterations in the total microRNA profiles of erythrocyte concentrates during storage and explore the viability of observing these changes post-transfusion. This innovative approach opens avenues for anti-doping technologies and commercialization, positioning it as a cornerstone in the ongoing fight against doping in sports and beyond. The significance of developing a robust detection method cannot be overstated, as it ensures the credibility of anti-doping efforts and promotes a level playing field for all athletes.

## 1. Historical Perspective of Doping and Stamina Boost Cause

Throughout history, individuals have sought various methods to gain an advantage over competitors in athletic pursuits [1]. In ancient times, this pursuit often involved the use of herbal mixtures and substances like bovine testicles, aligning with the original concept of “doping”, a term rooted in Afrikaans denoting stimulating concoctions [2]. Subsequently, amphetamine compounds and anabolic agents found deliberate application, with amphetamines enhancing alertness in soldiers and anabolic steroids aiding the recovery of malnourished prisoners of war [1]. The formal definition of doping emerged in 1963 when the Council of Europe characterized it as the use of foreign substances in unconventional ways to gain an unfair advantage in performance [3]. This definition, however, left room for ambiguity, as it did not cover naturally occurring hormones like testosterone, potentially contributing to its widespread misuse [4]. Surprisingly, the former German Democratic Republic even initiated state-sponsored doping initiatives to bolster its global sports reputation, even resorting to unethical human subject research, resulting in premature mortality for those involved [5]. In order to centralize global efforts in safeguarding athletes’ health and promoting fairness, the World Anti-Doping Agency (WADA) was established in 1999 following the inaugural worldwide anti-doping conference. One of its early actions was the adoption of “The Code”, which became effective in 2009 and underwent recent updates in 2021 [6]. This comprehensive and unifying document not only refines the definition of doping but also provides a comprehensive list of regulations and penalties for athletes who breach them. Under this regulatory framework, doping is defined as one or more breaches of the specified anti-doping rules. Banned materials considered detrimental to the spirit of sports, posing health risks, or having the potential to enhance athletic performance or mask its use, are outlined and regularly updated in the WADA’s “Prohibited List”, encompassing categories such as anabolic steroids, growth factors, β-2 agonists, hormone modulators, narcotic factors, gene-assisted doping, the handling of blood components, and so on [7].

The term “blood doping” typically encompasses the usage of substances like erythropoietin (EPO) stimulants, synthetic oxygen enhancers, blood substitutes, or blood transfusions, which are strictly forbidden under all circumstances [7]. The primary objective of blood doping is to improve oxygen delivery, which can be achieved by elevating factors like blood hemoglobin mass or oxygen transport capacity. This enhancement is crucial during aerobic exercise, and, as a result, it is particularly appealing to endurance athletes, although it may also have relevance to athletes in other disciplines [8]. For example, EPO is naturally synthesized in the kidneys under the regulation of hypoxia-inducible factors (HIFs) and plays a pivotal role in promoting the erythropoiesis of bone marrow (BM) [9]. However, the use of HIF stabilizers or synthetic human EPO (known as rhEPO) was originally intended to treat anemia [9]. Interestingly, these substances can significantly accelerate the production of red blood cells (RBCs), even to excess, in healthy individuals. Based on the half-life of rhEPO, athletes need to administer repeated injections of it, typically with an advanced preparation period of approximately one month before a competition. This timing is necessary to maximize the advantages of achieving peak hemoglobin concentration, given that erythropoiesis is a process that takes several weeks to unfold [10,11]. The misuse of rhEPO has additional repercussions beyond the augmentation of oxygen-carrying capacity. Firstly, it leads to the suppression of the body’s natural production of endogenous EPO as part of an effort to maintain internal equilibrium. Secondly, there is an elevated risk of thromboembolic complications due to the increased viscosity of the blood caused by the excess red blood cells produced as a result of rhEPO use [12].

In addition to its clinical purpose of raising the amount of hemoglobin in anemic patients, blood transfusions have also become a significant method of blood doping [13]. Although earlier practices involved the withdrawal and subsequent transfusion of whole blood, modern blood doping centers primarily around the targeted administration of specific blood components to minimize the volume load on the blood vessels [14]. In this procedure, blood components are segregated following withdrawal, and only the specific components of interest are transfused. When it comes to augmenting oxygen-carrying capacity, RBCs are the key functional unit. These RBCs can be preserved in suitable solutions at a temperature of 4 °C for a maximum of 49 days or cryopreserved for as long as 30 years, adhering to current regulatory guidelines [15].

Before a competition, the transfusion of stored RBCs results in an immediate elevation in the number of circulating RBCs and hemoglobin mass, which typically persists for 70 days, which aligns with the normal lifespan of RBCs, totaling 120 days, minus the duration of storage. Although red blood cell (RBC) transfusions substantially enhance physical performance, it is essential to acknowledge the accompanying adverse effects. Similar to the impact of rhEPO injections, elevated hematocrit levels result in increased blood viscosity, thereby elevating the risk of stroke, heart attack, and venous thromboembolism [16]. Additionally, immunological reactions need to be taken into account, particularly in the case of homologous or allogenic transfusions, whereas they might be less concerning in the context of autologous transfusions [17].

## 2. Detection of Blood Doping and Its Limitations

In 2009, the World Anti-Doping Agency (WADA) introduced the Athlete Biological Passport (ABP) as a means of continuous monitoring for athletes. The ABP employs Bayesian inference to assess an athlete’s unique physiological boundaries, predicting the likelihood of a marker falling within their normal range [18]. This passport comprises three modules: hematological, steroidal, and endocrinological. The hematological module, in particular, is designed to detect various forms of blood doping. The WADA strictly regulates the test parameters, as well as the procedures for sample collection, storage, transportation, and reporting, all of which are detailed in the WADA’s guidelines for ABP operation and sample collection [18,19]. The core indicators within the hematological module consist of hemoglobin levels and the OFF-hr score (=10 × hemoglobin − 60 × √ reticulocyte percentage) [20]. If an unusual finding in an athlete’s passport is detected, this occurs when one of the primary markers surpasses an individual’s 99% probability threshold, as established by the adaptive Bayesian inference model. In such cases, an expert panel conducts a thorough review of the results, taking into account additional available information. These supplementary data include longitudinal patterns of secondary markers (like reticulocyte percentage and abnormal blood profile score (ABPS)) and factors that could influence the markers (such as hematocrit levels, age, hydration status, whereabouts, etc.) [18]. Ultimately, the experts’ judgment can fall into one of the following categories: “normal”, “suspicious”, “likely doping”, or “likely medical condition.” Through this cautious approach, the WADA aims to safeguard nondoped athletes from unfounded accusations. However, this ethical stance may result in many athletes who are actually using prohibited substances going unpunished unless they are legitimately exposed by a testing method other than the athlete biological passport (ABP). In this context, more conclusive evidence regarding the misuse of prohibited substances or methods can be obtained through direct or indirect screening for external substances or their metabolites. In the unauthorized usage of rhEPO, the hematological module within ABP can merely confirm an escalation in erythropoiesis. However, a more definitive method for detecting the recombinant origin of the hormone in urine-based testing involves utilizing isoelectric focusing, which is visualized through immunoblotting. This method has received official approval as a means of detection [21]. Similarly, the presence of hemoglobin-based oxygen carriers can be reliably identified by employing a combination of chromatography and mass spectrometry [22]. Due to their stability within the body, perfluorocarbons can be directly detected either in exhaled air or blood, facilitated by gas chromatography-mass spectrometry (GC-MS) [23]. It is worth noting that there are currently 360 distinct erythrocyte antigens known, and the grouping of these antigens can differ from one individual to another, particularly in relation to the minor antigens [24]. Likewise, homologous infusions can be successfully accomplished using flow cytometric analysis. However, when it comes to autologous blood transfusions, this method is not applicable. There are promising approaches for detecting autologous blood transfusions based on measuring the total hemoglobin mass through the CO_2_ rebreathing procedure [25,26]. While it has been confirmed that an increase in total hemoglobin mass can be detected with high sensitivity and specificity after autologous blood transfusions, the main challenge lies in the method used to measure total hemoglobin mass itself. This measurement can be easily manipulated by the athlete, and furthermore, the use of carbon monoxide (CO) in this procedure carries potential risks for athletes [27]. As an alternative method, it has been demonstrated that plasticizers from blood bags and their breakdown products can migrate from the bag into the stored blood. This migration allows for their detection through liquid chromatography-mass spectrometry (LC-MS) in urine following a transfusion, even when plasticizer-free bags are used, resulting in lower concentrations [28]. Notably, research efforts that concentrate on the examination of hepcidin, along with the identification of immature reticulocytes using MS in dried blood spots, have yielded encouraging outcomes in the detection of autologous blood transfusions [29,30]. Nevertheless, there is currently no authorized technique available for definitively identifying autologous blood transfusions. Despite the demonstrated potential of the hematological parameters within the ABP to monitor changes associated with autologous blood transfusions, factors such as genetic variances, high-altitude training, the use of masking agents, and the timing of detection windows can significantly impact hematological profiles [31,32]. Notably, the anti-doping investigations are initiated not due to suspicious test results but rather rely on insider information for success, highlighting the pressing need for reliable testing methods to confirm autologous blood transfusions conclusively. In light of these challenges, it becomes evident that the development of dependable testing methodologies to definitively confirm autologous blood transfusions is an urgent and critical priority in the fight against doping in sports.

## 3. Transcriptomics as a Newcomer to the Field

**Figure 1:**
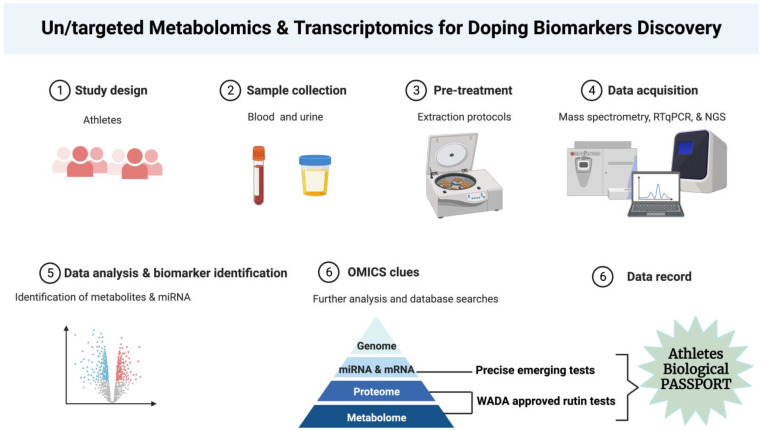
Untargeted/targeted metabolomics for the discovery of doping biomarkers.

Transcriptomics has emerged as a promising addition to the array of “omic”-based approaches commonly employed for disease detection, monitoring, and prognosis [33]. Transcriptomics has also captured considerable interest within the anti-doping domain. Durussel et al. utilized whole-blood mRNA profiling to pinpoint various genes influenced by microdosing with rhEPO, suggesting the potential of transcriptomics as a fresh dimension in ABPs [34]. Moreover, alterations in mRNA expression were observed in T-lymphocytes following autologous blood transfusions [35]. Notably, research in the realm of blood doping has extended beyond coding transcripts to include noncoding elements of the transcriptome. Leuenberger et al. identified significant changes in the levels of certain miRNAs following the use of erythropoiesis-inducing drugs [36]. Intriguingly, several miRNAs have previously been linked to alterations in erythropoiesis induced by autologous blood transfusions [37]. In building on these initial findings, two important factors come into play: first, erythrocytes are enriched with miRNAs that mirror the overall miRNA profile of whole blood [38] and second, erythrocytes exhibit highly significant changes in miRNA patterns during storage [39]. Haberberger et al. indicated that five miRNAs (miRNA-1260a, miRNA-1260b, miRNA-5100, miRNA-4443, and miRNA-4695-3p) were overexpressed in the long-term storage of EC [39]. Furthermore, exploring the dynamic changes in miRNA signatures in real-time post-transfusion provides a unique opportunity to enhance the precision of ABT detection within the ABP framework. The intricate analysis of these signatures holds promise in elevating the accuracy and reliability of anti-doping efforts, establishing a cutting-edge approach to safeguarding the integrity of competitive sports (Figure 1).

In the past, EVs were often regarded as the “cell’s waste disposal system”, primarily being responsible for eliminating unwanted cellular material. However, the contemporary understanding recognizes EVs as highly conserved, lipid bilayer-encased nanoparticles that cannot self-replicate. They are produced by nearly all cell types, exist in various biofluids, and play a significant role in cell-to-cell communication [40]. The term “EV” encompasses three main categories of vesicles: exosomes (less than 150 nm in size), microvesicles (up to 1000 nm), and apoptotic bodies (up to 5000 nm). While these vesicle types differ in size, density, and composition, they can also share common characteristics, making their biogenesis the most reliable distinguishing factor. Since determining the precise intracellular origin of these vesicles is challenging and can only be confirmed by tracking them at the moment of their release, the International Society for Extracellular Vesicles (ISEV) encourages the general use of the term “EV” [41].

EVs have emerged as essential players in the intricate landscape of health and disease. These nanoparticles are now recognized as key mediators in intercellular communication and are involved in a wide range of physiological processes [42]. Alterations in EV composition, cargo, and abundance have been associated with various diseases [43,44,45]. The remarkable potential of EVs lies in their ability to reflect specific disease signatures, offering a treasure trove of diagnostic and prognostic information [46,47,48]. Harnessing these changes in EVs for the early detection and monitoring of diseases has become an exciting frontier in medical research. By leveraging the evolving understanding of EV biology and developing innovative technologies for their isolation and analysis, scientists and clinicians are paving the way for more precise, non-invasive, and early disease detection strategies that hold great promise for improving patient outcomes and personalized medicine. The primary focus of this review is to provide an overview of the latest strategies for detecting autologous blood doping via EVs and miRNAs. It has been demonstrated that the storage of ECs significantly alters whole-blood miRNA profiles and increases the release of EVs carrying modified miRNA cargo [39,49,50]. As a result, investigations into both whole blood and EV-associated miRNAs have been conducted in vivo to explore their potential as biomarkers for uncovering autologous blood transfusions.

## 4. Comparing the Benefits: microRNA vs. Protein Biomarkers and Methods

There is an urgent need for the development of new miRNA and protein biomarkers in a range of applications in sports medicine and doping control that include diagnostics, disease stratification, therapeutic decisions, and preventing athlete’s doping abuse. One of the main classical technologies to address this need is the mass spectrometry method, which is used for protein biomarker discovery and, increasingly, also for protein biomarker validation. Data-dependent analysis, also known as shotgun proteomics and targeted mass spectrometric approaches, is now in application. Recently developed data-independent acquisition strategies combine the strengths of shotgun and targeted proteomics while avoiding some of the respective technologies’ limitations in the doping domain [51,52]. Recently, miRNA was introduced as a novel source of doping biomarker in sport medicine. However, there are pros and cons regarding protein- vs. miRNA-based approaches. For instance, this is made significantly difficult by the fact that most miRNA expression data in biofluids are expressed as relative expression values rather than absolute counts per volume. This affects data comparability not only between labs but also between studies from the same lab. While protein biomarkers are well established, even secondary metabolites can be detected precisely by mass spectrometry methods. On the other hand, the use of circulating miRNAs to detect performance-enhancing agents could also be extrapolated to other doping trends, such as testosterone abuse. Currently, the gold standard method of detecting exogenous testosterone administration is gas chromatography–mass spectrometry analyses of urine samples. In the study by Eklund et al. [53], 106 Olympic female athletes had considerably greater serum levels for dehydroepiandrosterone (DHEA) than sedentary control persons. The athletes’ serum levels for dihydrotestosterone and DHEA were positively associated with their physical performance. The above-mentioned issues question current mass spectrometry in terms of precisely evaluating these challenges. From that point of view, recent measurements demonstrate that the circulating miRNA-122 in the plasma and serum can be used to detect testosterone abuse precisely, and clinical results show that longitudinal measurements of miRNA levels in plasma could be a promising approach [54,55]. Furthermore, new evidence suggests that serum miRNAs are more stable at different temperatures than other sources of miRNA. This feature is critical because miRNAs might be used to detect illicit substance intake in the same manner that they are utilized as disease biomarkers [56,57]. When taken together, protein-based biomarkers are the gold standard in doping control; however, their limitation and poor sensitivity in ABT and other peptide-related doping facilitate miRNA usage as a new-generation biomarker in doping control (Figure 1).

## 5. Evaluation of the ABT-Induced miRNA Fingerprint in Blood

The detection of autologous blood transfusion misuse among high-performance athletes remains a formidable challenge. Traditional direct detection methods targeting exogenous substances administered to the body often fall short in this context. Instead, there is an urgent need for reliable indirect detection methods to align with the World Anti-Doping Agency’s (WADA) mission of ensuring both health and equality in sports. Currently, the per-individual longitudinal profiling of hematological parameters, as outlined in the athlete biological passport (ABP), stands as the primary approach for uncovering various forms of blood doping, including ABT. However, it is evident that a significant number of cases may go undetected, as highlighted by instances where athletes were only convicted based on evidence provided by whistleblowers. miRNAs have already demonstrated their potential in revealing other forms of doping, such as the misuse of anabolic agents, and, intriguingly, they undergo significant changes during erythrocyte storage. Mussack et al. [58] explored the capacity of miRNAs to identify individuals who have engaged in autologous blood transfusions, opening up new avenues for enhancing the detection of this prohibited practice. Their research revealed the considerable potential of miRNAs in detecting autologous blood doping, especially when analyzed six hours post-transfusion, achieving sensitivity levels of up to 11% at 100% specificity. While this may offer an extended detection window, it is important to note that the classification performance of miRNA patterns still fell short of the current gold standard ABP, which achieved higher sensitivity rates of up to 44% at 100% specificity, with the highest sensitivity observed two days after transfusion [58]. The further investigation and refinement of miRNA signatures could potentially lead to their integration into routine anti-doping tests, ultimately determining their true utility in the ongoing battle against blood doping.

The number of studies focusing on miRNA as a potential biomarker for blood doping is limited (Table 1). Among these, only three studies specifically examined the application of ABT involving the use of stored refrigerated or cryopreserved ECs or blood [37,58,59]. Leuenberger et al. discovered three miRNAs (miRNA-26b, miRNA-30b, and miRNA-30c) that exhibited distinct regulation patterns up to one day after ABT following 42 days of erythrocyte concentrate storage [59]. Gasparello et al. conducted an investigation into miRNA changes (miRNA-92a-3p miRNA-126-3p, miRNA-144-3p, miRNA-191-3p, miRNA-197-3p, miRNA-486-3p, and miRNA-486-5p) 15 days after the autologous retransfusion of erythrocyte concentrate that had been stored for 35 days [37], revealing the significant overexpression of miR-197-3p in athletes who had undergone ABT. However, this time frame might be considered too late to effectively capture significant ABT-induced effects during competitive events for a couple of reasons. Firstly, it is believed that the most significant impact of ABT on exercise performance and, consequently, an athlete’s miRNA pattern occurs within the initial two to three days following transfusion [60]. Secondly, athletes attempting to deceive doping tests are known to receive EC transfusions on the day of a competition and then promptly donate blood shortly afterward to eliminate the hematological enhancements before their next doping test. By accounting for these factors and selecting appropriate sampling time points, Mussack et al. successfully identified an ABT-induced miRNA fingerprint (miRNA-144-3p and miRNA-320d) through comprehensive miRNA screening, as determined by DGE and sPLS-DA patterns [58]. These miRNAs act similarly and have been explained as erythropoiesis-inducers [61,62]. The increased expression of miRNA-144 following treatment with EPO-stimulating agents further supports this connection, which could be applied as a promising biomarker to uncover erythropoiesis-stimulating ingredients in anti-doping [36]. The notion is supported by the decline in miR-144-3p and miR-144-5p expression levels after blood donation, followed by their prompt restoration upon retransfusion, with both miRNAs being abundant in erythrocytes and pivotal in erythroid differentiation [38,63,64]. Furthermore, these results reinforce the idea that miRNAs originating from stored ECs could exert a substantial and immediate impact on the circulating miRNA profile upon retransfusion. Consequently, it is reasonable to speculate that miRNAs identified as markedly upregulated during extended EC storage would also rapidly appear in substantial quantities in the circulation following retransfusion. Interestingly, none of the miRNAs differentially regulated upon ABT were identified in these three studies because of exploring different biofluids.

As demonstrated by the OFF-hr score and ABPS, combining multiple indicators can improve the detection of doping [20,66]. In the quest for additional data to enhance the detection of ABT, beyond relying solely on hematological markers and total blood miRNAs, urine presents an overlooked biospecimen. Urine samples are routinely gathered in anti-doping controls and are authorized for detecting rhEPO [67]. Physical activity and an individual’s overall physical condition can influence blood volume and potentially alter total blood miRNA profiles due to exercise-induced leukocytosis [68]. However, urinary miRNA patterns are expected to remain largely unaffected by these shifts in cellular composition. Furthermore, urinary miRNAs carried by EVs are highly stable [69], making them potentially robust biomarkers for detecting ABT. The particular interest in urinary EV-associated miRNA signatures for ABT detection arises from the fact that the cold storage of ECs leads to both an increase in EV concentration and significant alterations in miRNA expression within these EVs [49,50]. Theoretically, EVs are considered too large to be filtered by the nephron in healthy humans [70,71]. Nevertheless, experiments in mice have demonstrated the trans-renal release of labeled EVs previously introduced into the circulation [72]. Furthermore, Mussack et al. observed a notable rise in urinary EV concentrations shortly after the retransfusion of ECs in humans [73], supporting the idea of transfused EVs being excreted in urine. Intriguingly, recent research by Mussack et al. has shown that at least some urinary EVs indeed bear surface markers indicative of a hematological origin [73]. Despite these promising findings, investigating urinary EVs for their potential as biomarkers presents both biological and technical challenges that may impact reproducibility and comparability, necessitating careful consideration.

## 6. Effecting Factors to the miRNA Pattern

The miRNA pattern can be influenced by various factors that play a crucial role in shaping its dynamics and implications. For example, smoking impacts miRNA profiles significantly and should be considered as playing a role in clinical trials [74]. Similarly, the hypoxic conditions experienced at high altitudes also bring about changes in the miRNA patterns found in erythrocytes and hematological markers [63]. Additionally, hormonal fluctuations due to the menstrual cycle in females impact the hematological parameters incorporated in the ABP [75]. Likewise, the widely defined population-based reference range for a “normal” hematocrit value fails to distinguish an individual’s abnormal value. Moreover, significant variations exist in the hematological parameters among individuals, potentially concealing individual outliers within the average differences. Therefore, it is crucial to longitudinally monitor individual profiles and establish specific reference ranges for each person. According to Aikin et al., acquiring three samples over three to four weeks adequately monitors intra-individual variations in the doping biomarkers influenced by external factors like diet, hydration, and physical activity [76]. The storage of blood is another main factor that should be considered as well. In order to detect ABT, the stored blood bags should be reinfused into the donor to boost their erythrocyte levels. Identifying dopped sinners based on distinct miRNA expression profiles relies solely on highly upregulated miRNAs. It is worth mentioning that unsuitable miRNAs as biomarkers should be excluded because of their natural arbitrary variation in physiologic conditions. Haberberger et al. introduced six ‘unstable miRNAs’ (let-7g-5p, miRNA-126–3 p, let-7a-5p, let-7i-5p, miRNA-107, and miRNA-107) over two weeks of storage [39]. Furthermore, they indicated that during blood storage, 189 miRNAs were stable over two weeks of storage, and 28 miRNAs were altered due to processing, which could serve as a detection alarm for ABT [39]. Surprisingly, there was a notable contrast in the levels of miRNA-144-3p between the samples collected right before blood processing into ECs and the samples taken from the freshly prepared ECs, suggesting that the transition of blood into a storage bag and its subsequent processing into ECs holds more relevance for doping detection than the storage period itself. Consequently, this extends the detection window to an earlier phase, which is in contrast to the ABP (where the ABT detection window typically spans one to two days post-EC retransfusion). Currently, the only sanctioned sample matrices in doping controls include serum, EDTA blood, and urine. The stringent regulations govern their collection, transportation, analysis, and storage, primarily focusing on their stability during transit and storage and minimizing the possibility of tampering [19]. Notably, minimally invasive sampling methods are favored. Within this scope, emerging sample matrices, such as dried blood spots, are being considered for additional testing material due to their easy collection and resistance to the changes induced during transport [29]. Whole-blood miRNA profiles, obtained using PAXgene Blood RNA tubes, exhibit notable stability and limited susceptibility to the alterations caused by transportation and temperature variations, suggesting their potential use as new test materials in doping controls [58]. These findings suggest that the intra-individual factors and storage-related modifications should be brought to the matter while detecting ABT using miRNAs.

Additionally, it is worth discussing the potential challenges posed by the lack of nuclei in erythrocytes, which traditionally limits their ability to transcribe genetic material. The observed changes in miRNA patterns during storage in erythrocytes can be attributed to several factors, as follows: while erythrocytes lack nuclei, they release *extracellular vesicles*, such as exosomes, which carry miRNAs. These vesicles serve as messengers, allowing erythrocytes to communicate with other cells and tissues [77,78]. Therefore, alterations in miRNA patterns during storage may be linked to changes in the release of these vesicles. The concentration of EVs in erythrocyte concentrates is affected by component preparation methods, storage conditions and solutions, the leukoreduction method, and inter-donor variation, as comprehensively reviewed by Wannez et al. [79]. The storage conditions of blood involve various environmental factors, such as temperature, pH, and anticoagulants, which can impact the stability of miRNAs [80], as we discussed earlier. Similarly, despite the lack of nuclei in mature erythrocytes, the blood storage process may involve contamination from residual nucleated cells [81]. These cells can contribute to miRNA changes, and their presence should be considered in the context of storage-related alterations. Likewise, factors such as RNase activity [82], temperature fluctuations (freezing, thawing, and long-term storage) [83], and the presence of stabilizing proteins [84,85] may influence miRNA stability. Moreover, while erythrocytes are primarily known for miRNA content, the potential role of long noncoding RNAs (lncRNAs), particularly those acting as sponges, has not been investigated yet. These molecules may interact with miRNAs, affecting their availability and functionality. Investigating the interplay between miRNAs and sponging lncRNAs can shed light on the regulatory mechanisms at play during storage. By delving into the stability of miRNAs, a comprehensive understanding of the molecular dynamics underlying autologous blood doping in athletes could be offered.

## 7. Conclusions 

In summary, the pressing need for robust detection methodologies to identify ABT in athletes has led to a growing focus on miRNAs as potential biomarkers. The insidious nature of ABT doping in competitive sports necessitates precise and reliable detection methods to ensure fairness and integrity. By delving into the intricate world of miRNA patterns and exploring their alteration during blood storage and post-transfusion, our review highlights the promising role of miRNAs in detecting ABT. 

## 8. Future Perspectives

This innovative field presents a unique opportunity for the development of novel anti-doping technologies and commercial prospects, which are essential in combatting doping in sports. Leveraging miRNAs as silent informants in the blood offers a potential pathway to fortify the fight against ABT, contributing significantly to the integrity and fairness of competitive sports while paving the way for advancements in anti-doping measures. While RNA quantification is currently not endorsed by the accredited labs of the WADA, there is no apparent barrier preventing this from evolving in the future. This transition is justifiable since quantitative PCR (qPCR) is a readily standardizable method routinely applied in clinical labs, characterized by its cost-effectiveness and minimal personnel requirements.

## Figures and Tables

**Table 1:** Research investigations in anti-doping, focusing on exploring miRNAs as potential biomarkers for the detection of blood doping.

Type of Blood Doping	Species	Expressed miRNAs		Ref.
		Up	Down	
**ABT**	Human	miR-197-3p	-	[37]
**ABT**	Human	miRNA-26a, miRNA-26b, miRNA-30b, miRNA- 30c, miRNA-103, miRNA-142-3p, miRNA-339-5p, let-7b, let-7d, let-7g	-	[59]
**ABT**	Human	miRNA-144-3p	miRNA-320d	[58]
**rhEPO**	Human	miRNA-17, miRNA-19a, miRNA-19b, miRNA-25, miRNA-29c, miRNA-92a, miRNA-93, miRNA-101, miRNA-106b, miRNA-140-3p, miRNA-142-5p, miRNA-144, miRNA-185	miRNA-874	[36]
**HIF booster**	Rat	miRNA-21, miRNA-130a	-	[65]

ABT: autologous blood transfusion; rhEPO: recombinant human erythropoietin; HIF: hypoxia-inducible factor.

## Data Availability

Not applicable.
